# A novel endonuclease that may be responsible for damaged DNA base repair in *Pyrococcus furiosus*

**DOI:** 10.1093/nar/gkv121

**Published:** 2015-02-18

**Authors:** Miyako Shiraishi, Sonoko Ishino, Takeshi Yamagami, Yuriko Egashira, Shinichi Kiyonari, Yoshizumi Ishino

**Affiliations:** Department of Bioscience and Biotechnology, Graduate School of Bioresource and Bioenvironmental Sciences, Kyushu University, 6-10-1 Hakozaki, Higashi-ku, Fukuoka, Fukuoka 812-8581, Japan

## Abstract

DNA is constantly damaged by endogenous and environmental influences. Deaminated adenine (hypoxanthine) tends to pair with cytosine and leads to the A:T→G:C transition mutation during DNA replication. Endonuclease V (EndoV) hydrolyzes the second phosphodiester bond 3′ from deoxyinosine in the DNA strand, and was considered to be responsible for hypoxanthine excision repair. However, the downstream pathway after EndoV cleavage remained unclear. The activity to cleave the phosphodiester bond 5′ from deoxyinosine was detected in a *Pyrococcus furiosus* cell extract. The protein encoded by PF1551, obtained from the mass spectrometry analysis of the purified fraction, exhibited the corresponding cleavage activity. A putative homolog from *Thermococcus kodakarensis* (TK0887) showed the same activity. Further biochemical analyses revealed that the purified PF1551 and TK0887 proteins recognize uracil, xanthine and the AP site, in addition to hypoxanthine. We named this endonuclease Endonuclease Q (EndoQ), as it may be involved in damaged base repair in the *Thermococcals* of Archaea.

## INTRODUCTION

DNA is damaged by endogenous and environmental influences. Extensive studies on excision repair systems, including nucleotide excision repair and base excision repair (BER), are being performed around the world, and our knowledge is constantly increasing (1–4). The excision repair initiated by a single nick near the site of a DNA lesion is now referred to as alternative excision repair (AER). This type of repair starts with an endonuclease that recognizes the damaged DNA and cleaves the phosphodiester bond near the lesion site (5–7).

Base deamination is a typical form of DNA damage. Deaminated adenine, guanine and cytosine are called hypoxanthine, xanthine and uracil, respectively. These deaminations occur spontaneously under physiological conditions, and are promoted by ionizing radiation, high temperature, aerobic respiration and nitrosative stress. The hydrogen bonding properties of the bases are altered by the amino-keto conversion derived from deamination. For example, hypoxanthine in DNA tends to pair with cytosine, but not thymine, which is the natural binding partner of adenine. This property of hypoxanthine leads to an A:T→G:C transition mutation during DNA replication (8). Therefore, the hypoxanthine sites must be repaired to prevent mutations.

Two major pathways, BER and AER, are known to remove the deaminated bases. The BER pathway is based on DNA glycosylase, and several enzymes belonging to the uracil DNA glycosylase (UDG) superfamily have been identified (9). On the other hand, AER is initiated by nicking at the lesion site by a specific endonuclease (5). Endonuclease V (EndoV) is well known as the enzyme responsible for cleaving the second phosphodiester bond on the 3′-side of the deaminated base lesion. EndoV, encoded by the *nif* gene, was originally identified in *Escherichia coli* as an endonuclease that nicks DNA containing a damaged base, and was subsequently proved to be a deoxyinosine (dI) 3′-endonuclease (10–12). Furthermore, analyses of *nif* mutant strains revealed that *E. coli* EndoV plays a major role in dI repair in the cells, although broader substrate specificity toward mismatched base pairs, including apurinic/apyrimidinic (AP) sites, flap DNA and pseudo-Y DNA structures, was detected *in vitro* (12–14).

EndoV homologs are conserved in all three domains of life: Bacteria, Eukarya and Archaea (15,16). The endonuclease activity for DNA containing dI has been shown for the mouse and human enzymes, as the eukaryotic EndoVs (17,18). However, it has not been determined whether the AER pathway with the EndoV homolog actually functions in eukaryotic cells. The archaeal EndoVs are diverse. *Archaeoglobus fulgidus* EndoV (AfuEndoV) exhibits strict specificity for dI-containing substrates *in vitro* (19). On the other hand, the *Ferroplasma acidarmanus* enzyme consists of the O^6^-alkylguanine-DNA alkyltransferase domain and the EndoV domain (therefore, it is called FacAGT-EndoV), and shows cleavage activities for DNA substrates containing uracil, hypoxanthine and xanthine bases *in vitro* (20). We characterized the EndoV homolog from the hyperthermophilic euryarchaeon, *Pyrococcus furiosus* (PfuEndoV) and discovered its strict substrate specificity to hypoxanthine *in vitro* (21). To elucidate the EndoV-mediated repair pathway in archaeal cells, the proteins related to the cleavage reaction of dI-containing DNA were screened, and we identified the protein possessing the activity to cleave the phosphodiester bond 5′ from dI. This novel endonuclease, designated as Endonuclease Q (EndoQ), is conserved only in *Thermococcals* and some of the methanogens in Archaea, and is not present in most Bacteria and Eukarya.

## MATERIALS AND METHODS

### DNA substrates

The 7-deaza-2′-deoxyxanthosine (dX)-containing oligonucleotide was obtained by custom synthesis (BEX, Tokyo, Japan). The other oligonucleotides, including the dI, deoxyuridine (dU) and tetrahydrofuran (AP)-containing oligonucleotides, were obtained from Hokkaido System Science (Sapporo, Japan) and Sigma Genosys (Tokyo, Japan). The tetrahydrofuran-containing oligonucleotide (45-AP25) was used as a model compound of the AP site. Fluorescently (Cy5 or FITC) or ^32^P-labeled oligonucleotides were synthesized or prepared, respectively. Double-stranded DNA was prepared by incubating the oligonucleotide and its complementary oligonucleotide in TAM buffer (40-mM Tris-acetate, pH 7.8, and 0.5-mM magnesium acetate) and annealing by an incubation in a decreasing temperature gradient. The sequences of the oligonucleotides and the oligonucleotide pairs for dsDNA are shown in Supplementary Table S1.

### *P. furiosus* cell cultivation and cell extract fractionation

*P. furiosus* was cultivated with agitation at 98°C in 4 l of medium, containing 10-g Bacto Tryptone (BD), 5-g Bacto Yeast Extract (DIFCO), 38-g Marine Art SF-1 (Osaka Yakken; consisting of 22.1-g NaCl, 9.9-g MgCl_2_, 1.5-g CaCl_2_·2H_2_O, 3.9-g Na_2_SO_4_, 0.61-g KCl, 0.19-g NaHCO_3_, 96-mg KBr, 78-mg Na_2_B_4_O_7_·10H_2_O, 13-mg SrCl_2_, 3-mg NaF, 1-mg LiCl, 81-μg KI, 0.6-μg MnCl_2_·4H_2_O, 2-μg CoCl_2_·6H_2_O, 8-μg AlCl_3_·6H_2_O, 5-μg FeCl_3_·6H_2_O, 2-μg Na_2_WO_4_·2H_2_O and 18-μg (NH_4_)_6_Mo_7_O_24_·4H_2_O), and 10-g Soluble Starch (Nacalai Tesque) per liter. The cells were grown to an OD_600_ of 0.58. After cultivation, the cells were harvested by centrifugation (3315 × *g*, for 5 min at 25°C) and sonicated for 5 min in buffer A (20-mM potassium phosphate, pH 7.4, 0.1-M NaCl, 0.5-mM DTT, 0.1-mM ethylenediaminetetraacetic acid (EDTA) and 10% glycerol) containing Complete^TM^, EDTA-free Protease Inhibitor Cocktail Tablets (Roche). The soluble cell extract obtained by centrifugation (10 min, 24 000 × *g*, 4°C) was subjected to phosphocellulose (P11) chromatography (Whatman). The bound proteins were eluted with a linear gradient of 0.1–1.0-M NaCl in buffer A. The fractions containing the cleavage activity on the 5′ side of dI within the 49-I25 dsDNA were dialyzed against buffer B (50-mM Tris-HCl, pH 8.0, 0.5-mM DTT, 0.1-mM EDTA and 10% glycerol) containing 0.1-M NaCl. The solution was then applied to a 1-ml HiTrap SP HP column (GE Healthcare) and eluted with a linear gradient of 0.1–1.0-M NaCl in buffer B. The fractions containing the cleavage activity were diluted with buffer B, to adjust the salt concentration to 0.1-M NaCl. The solution was further applied to a 1-ml HiTrap Heparin HP column (GE Healthcare), and eluted with a linear gradient of 0.1–1.0-M NaCl in buffer B. The fractions containing the cleavage activity were dialyzed against buffer C (10-mM KH_2_PO_4_/K_2_HPO_4_, pH 7.4). The solution was applied to a 1-ml Hydroxyapatite column (ECONOPACK CHT-II; Bio-Rad) and eluted with a linear gradient of 0.01–0.5-M KH_2_PO_4_/K_2_HPO_4_ in buffer C. The fractions containing the cleavage activity were dialyzed against buffer B. The solution was then applied to a 1-ml MonoS HR 5/5 column (GE Healthcare) and eluted with a linear gradient of 0–0.3-M NaCl in buffer B. All fractions were analyzed by 12% sodium dodecyl sulphate-polyacrylamide gel electrophoresis (SDS-PAGE) at every chromatography step. Fraction 48 from the MonoS chromatography was analyzed by 10% SDS-PAGE. The fractions with the cleavage activity were subjected to a mass spectrometry (MS) analysis.

### Cleavage assay of dI-containing DNA for screening

The cleavage reactions were performed at 60°C for 1 h in a 20-μl reaction mixture, containing 5-nM 49-I25 dsDNA and 2-μl fraction or 10-nM EndoV in reaction buffer (50-mM Tris-HCl, pH 8.0, 1-mM DTT, 1-mM MgCl_2_ and 0.01% Tween 20). After the incubation, 50-μg/ml Proteinase K (Nacalai Tesque) and 0.2% SDS were added to the reaction mixtures and incubated at 50°C for 30 min. The reactions were terminated by adding 5-μl stop buffer (98% formamide, 10-mM EDTA and 0.1% Orange G). After an incubation at 98°C for 5 min, the samples were immediately transferred to ice. The samples were separated by 8-M urea-12% PAGE in TBE buffer (89-mM Tris, 89-mM boric acid and 2.5-mM EDTA, pH 8.3). The gel image was visualized by using an image analyzer, Typhoon Trio+ (GE Healthcare).

### Cleavage assay using recombinant proteins

The cleavage reactions were performed at 75°C in a 20-μl reaction mixture, containing 50-mM Tris-HCl, pH 8.0, 1-mM DTT, 1-mM MgCl_2_, 0.01% Tween 20, 10-nM DNA substrate and 20-nM PF1551 or TK0887. The reactions were terminated and the samples were assayed, according to the aforementioned procedure.

### Identification of proteins by MS

The protein fraction, purified by sequential column chromatography steps, was subjected to SDS-PAGE, and each silver-stained band was excised, treated with trypsin, and analyzed by high-sensitivity LC/MS at the Laboratory for Technical Support, Institute of Bioregulation, Kyushu University, by using a Paradigm MS4 HPLC (Michrom Bioresources) and an LTQ mass spectrometer (Thermo Fisher Scientific). The proteins included in each band were identified by using the MASCOT search engine. The proteins with MASCOT scores >100 were considered as candidates.

### Cloning of the genes encoding PF1551 from *P. furiosus* and TK0887, the homolog of PF1551, from *Thermococcus kodakarensis*

The gene encoding the hypothetical protein PF1551 was amplified by polymerase chain reaction (PCR) directly from *P. furiosus* genomic DNA, using the forward primer PF1551-F (5′-GGGCCATG GTAGTTGATGGCGATCTGCACA-3′, the NcoI restriction site is underlined) and the reverse primer PF1551-R (5′-GGGGCGGCCGCTTAATTTACCTCTTTATTTTT AATATATTGAAGC-3′, the NotI restriction site is underlined). The PCR reaction mixture (50 μl) consisted of 80 ng of *P. furiosus* genomic DNA, 400 nM each of the forward and reverse primers, 0.2 mM of each dNTP and 2.5 units of Pfu DNA polymerase (Agilent), in 20-mM Tris-HCl, pH 8.0, 2-mM MgCl_2_, 10-mM KCl, 10-mM (NH_4_)_2_SO_4_, 0.1% Triton X-100 and 0.1-mg/ml bovine serum albumin. The amplified gene was digested by NcoI (New England Biolabs) and NotI (New England Biolabs) and ligated by T4 DNA ligase (New England Biolabs) into the corresponding sites of the pET21d expression vector (Novagen). The resultant plasmid was designated as pET-PF1551, and its nucleotide sequence was confirmed by sequencing. The gene encoding the hypothetical protein TK0887 was directly amplified by PCR from *Thermococcus kodakarensis* genomic DNA, using the forward primer TK0887-F (5′-CGCGCATATGATCGTTGATGCCGACCTGCAC-3′, the NdeI restriction site is underlined) and the reverse primer TK0887-R (5′-GGGGCGGCCGCTTACTTATTTGACTTTCGGAGGAATTCGG-3′, the NotI restriction site is underlined). The PCR reaction was exactly the same as that for PF1551. The amplified gene was digested by NdeI and NotI and ligated into the corresponding sites of the pET21a expression vector (Novagen). The resultant plasmid was designated as pET-TK0887, and its nucleotide sequence was confirmed by sequencing.

### Overproduction and purification of PF1551 and TK0887

PF1551 was overproduced in *E. coli* BL21 CodonPlus (DE3)-RIL (Agilent) cells carrying pET-PF1551, grown with shaking in 1 l of LB medium, containing 50-μg/ml ampicillin and 34-μg/ml chloramphenicol, at 37°C until the culture attained an OD_600_ of 0.4. IPTG was then added to a final concentration of 0.1 mM, and the cells were further grown for 24 h at 18°C to induce the expression from pET-PF1551. The cells were harvested by centrifugation (10 min, 5000 × *g*, 4°C) and sonicated for 10 min in buffer D (50-mM Tris-HCl, pH 8.0, 0.5-mM DTT, 1-mM EDTA and 10% glycerol) containing 0.1-M NaCl and 1-mM PMSF. The soluble cell extract obtained by centrifugation (10 min, 24 000 × *g*, 4°C) was heated at 80°C for 30 min. The heat-resistant fraction obtained by centrifugation (10 min, 24 000 × *g*, 4°C) was treated with 0.15% PEI, in order to remove nucleic acids. The soluble fraction obtained by centrifugation (10 min, 24 000 × *g*, 4°C) was precipitated by 80% saturated ammonium sulfate. The precipitate was obtained by centrifugation (10 min, 24 000 × *g*, 4°C) and then resuspended in buffer D containing 1.5-M (NH_4_)_2_SO_4_. The soluble fraction was applied to a 5-ml HiTrap Phenyl HP column (GE Healthcare) and eluted with a linear gradient of 1.5–0-M (NH_4_)_2_SO_4_ in buffer D. The fractions containing PF1551 were dialyzed against buffer D, containing 0.1-M NaCl overnight at 4°C, and were applied to a 1-ml HiTrap Heparin HP column (GE Healthcare). The column was developed with a linear gradient of 0.1–1.0-M NaCl in buffer D. The fractions containing PF1551 were diluted with buffer D, to adjust the salt concentration to 0.1-M NaCl, and applied to a 1-ml HiTrap SP HP column (GE Healthcare). The column was developed with a linear gradient of 0.1–1.0-M NaCl in buffer D. The protein fraction showing the activity was stored at −25°C, after the addition of 50% glycerol. The expression and purification of TK0887 were performed in basically the same manner as for PF1551. The protein concentration was calculated by measuring the absorbance at 280 nm. The theoretical molar extinction coefficient of both proteins is 47 120.

### Western blot analysis

*Thermococcus kodakarensis* cells (4.5 × 10^11^ cells) were disrupted by sonication in 15 ml of buffer, containing 50-mM Tris-HCl, pH 7.0, 0.5-mM DTT, 0.1-mM EDTA and 10% glycerol with proteinase inhibitor (Complete^TM^, Roche Diagnostics GmbH), and the extract was obtained by centrifugation. The cell extract and the purified recombinant TK0887 protein were separated by 12% SDS-PAGE, electro-blotted onto PVDF membrane (Bio-Rad) and reacted with anti-TK0887 antiserum, which was prepared by immunizing a rabbit with the recombinant TK0887 protein. The protein bands were detected by using ECL Prime (GE Healthcare) and quantified by LAS-3000mini image analyzer (FUJIFILM).

### Electrophoresis mobility shift assay

Various concentrations (0, 2.5, 5, 12.5, 25 and 50 nM) of PF1551 were incubated with 5-nM 5′-Cy5-labeled 45-I25 ssDNA or dsDNA, in a reaction solution (50-mM Tris-HCl, pH 8.0, 1-mM MgCl_2_, 1-mM DTT and 0.01% Tween 20) at 60°C for 10 min. After 5 μl of loading buffer (16.5% Ficoll, 22-mM Tris-HCl, pH 8.0 and 0.1% Orange G) was added, 5-μl portions of the samples were subjected to 4% PAGE in TBE buffer and visualized by an image analyzer, Typhoon Trio+ (GE Healthcare).

### Re-ligation of the TK0887 cleavage products

The cleavage products were prepared by an incubation of reaction mixtures (50-mM Tris-HCl, pH 8.0, 1-mM DTT, 1-mM MgCl_2_, 0.01% Tween20 and 15-nM 45-I25 dsDNA or 45-U24 dsDNA) at 75°C for 20 min in a 50 μl with or without 10-nM TK0887. Reactions were terminated by incubation at 98°C for 10 min. The cleavage products were annealed by gradually cooling to 25°C. Exchange of the reaction solution to water was performed using Illustra MicroSpin G-25 Column (GE Healthcare). The annealed DNA was subjected to ligation by Quick Ligation Kit (New England Biolabs) at room temperature for 30 min. The samples (1 μl) were separated by 8-M urea-12% PAGE in TBE buffer. The gel image was visualized by the image analyzer, Typhoon Trio+ (GE Healthcare).

## RESULTS

### Identification of the activity to cleave dI-containing DNA in *P. furiosus* cell extracts

**Figure 1.**
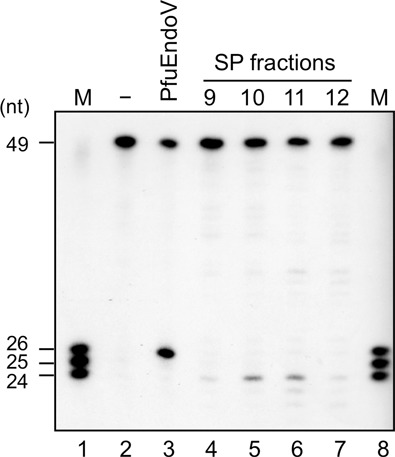
Cleavage activity of *P. furiosus* cell extracts toward dI-containing DNA. 5′-^32^P-labeled 49-I25 dsDNA (5 nM) was incubated at 60°C for 1 h with no enzyme (lane 2), 10-nM PfuEndoV (lane 3) and aliquots of SP column fractions from *P. furiosus* cell extracts (lanes 4–7), in the reaction solution described in the Materials and Methods section. The product markers (mixture of 24, 25 and 26 nt) are shown in lanes 1 and 8.

Our previous work showed that *P. furiosus* EndoV remains bound to the DNA after cleaving the second phosphodiester bond on the 3′-side of the dI (21). This result motivated us to search for another factor related to the cleavage activity of the dI-containing DNA, in the total extract of *P. furiosus* cells. However, we were not successful. Therefore, we fractionated the extract by cation-exchange chromatography, because many proteins in *P. furiosus* cells are negatively charged at neutral pH. EndoV was previously identified in *P. furiosus*, and was reportedly retained by a resin for the cation-exchange chromatography (21). Therefore, its activity should be detectable somewhere in the fractions. However, the detected cleavage product was two nucleotides shorter than that of EndoV, indicating that the cleavage site is on the 5′-side of the dI site (Figure 1). No endonuclease that directly cleaves on the 5′-side of the dI has been reported yet, and we tried to identify the protein corresponding to this activity. The western blot analysis using the anti-PfuEndoV antibody showed that PfuEndoV was also detected in the same fractions as the new activity (data not shown). The substrate DNA for this cleavage assay was labeled at its 5′-end, and therefore, the EndoV-cleavage product was probably not detected because the novel activity worked simultaneously with or after the EndoV cleavage reaction.

### Identification of the protein possessing the activity to cleave the dI-containing DNA on the 5′-side

**Figure 2.**
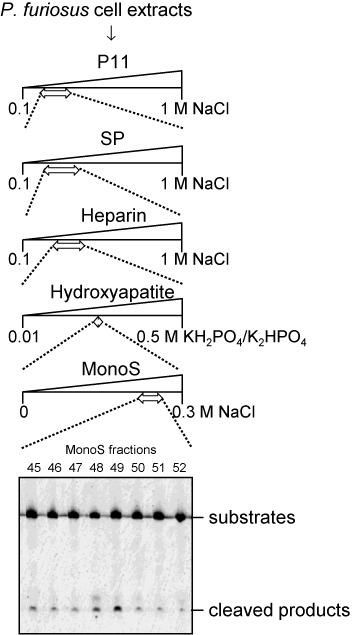
Purification scheme for the enzyme exhibiting cleavage activity on dI-containing DNA. *Pyrococcus furiosus* cell extracts (from 4 l of culture) were fractionated on five kinds of columns: P11, SP, Heparin, Hydroxyapatite and MonoS. An aliquot of each fraction was assayed for its activity on 5′-Cy5-labeled 49-I25 dsDNA. The fractions indicated by arrows showed the cleavage activity, and were subjected to downstream purification. The cleavage products were separated by 8-M urea-12% PAGE.

To concentrate the target activity, sequential chromatography steps were performed. First, the *P. furiosus* cell extract was applied to a phosphocellulose (P11) column, and the active fraction that eluted at 0.20–0.37 M NaCl was subjected to HiTrap SP cation-exchange column chromatography. The active fractions that eluted at 0.21–0.41-M NaCl were separated by a HiTrap Heparin column, and the active fraction that eluted at 0.27–0.46-M NaCl was applied to the Hydroxyapatite column. The activity that eluted at 0.23–0.26-M potassium phosphate was subjected to further cation-exchange chromatography (MonoS). These sequential chromatography steps and the results of the cleavage assay are shown in Figure 2. The proteins included in fractions 48 and 49, which showed the strongest cleavage activity, were analyzed by SDS-PAGE, but these fractions still contained many proteins with various sizes.

### MS analysis of the purified fraction

**Figure 3.**
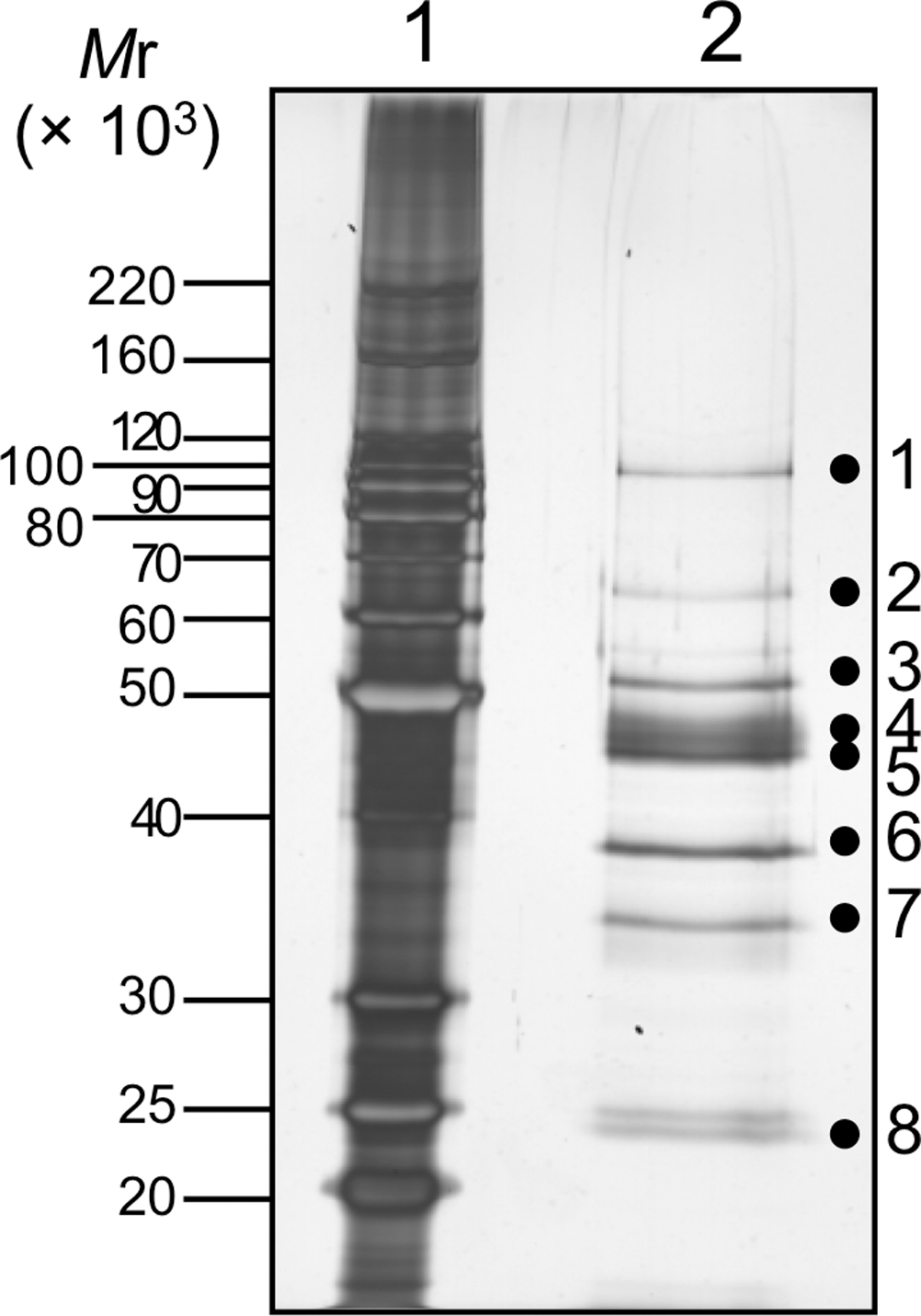
SDS-10%PAGE analysis of fraction 48 from the MonoS-column chromatography, followed by silver staining. The sizes of markers (lane 1) are shown on the left side of the panel. The proteins in fraction 48 (lane 2) were cut into eight slices, as shown by the dots on the gel, and were subjected to the MS analysis.

To identify the proteins included in the purified fraction, the silver-stained bands on the SDS-PAGE gel were excised and subjected to the MS analysis. Eight different bands were analyzed, and 19 proteins were identified as the candidates for the novel endonuclease, as shown in Supplementary Table S2. Based on comparisons of the respective band intensities with the activity strengths, the annotations of the proteins, and the conservation of amino acid sequences in Archaea, the hypothetical proteins PF0988 (AAL81112.1, Figure 3, slice 8), PF1551 (AAL81675.1, slice 4 and 5) and PF1862 (AAL81986.1, slice 8) became the leading candidates. The gene encoding PF0988 is located downstream of the gene encoding EndoV (PF0987) on the *P. furiosus* genome. The gene encoding PF1551 is annotated as a hypothetical protein and has a polymerase and histidinol phosphatase (PHP) domain-like sequence. PF1862 is annotated as a DNA-binding protein, and belongs to a family of several uncharacterized archaeal proteins. These genes were cloned and expressed in *E. coli*. Cell extracts of the *E. coli* transformants producing these recombinant proteins were incubated at 80ºC for 20 min to inactivate the *E. coli* proteins, and then subjected to the endonuclease assay, which revealed that PF1551 is the corresponding protein. The recombinant PF1551 protein was purified to homogeneity (Supplementary Figure S1) and was used for further characterization. PF1551 consists of 424 amino acids (MW 47 653.0).

### Cleavage property of the detected protein

**Figure 4.**
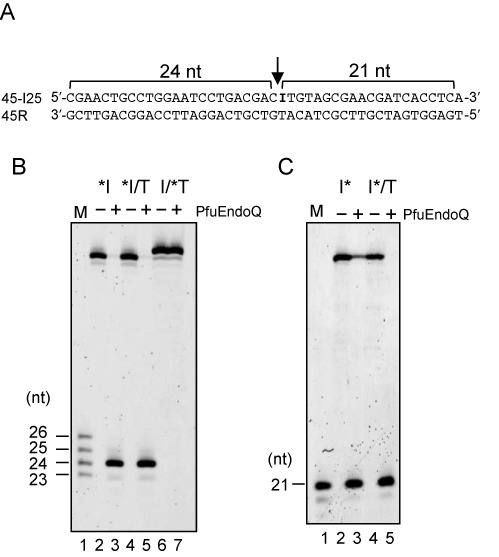
Cleavage properties of PfuEndoQ. (**A**) The nucleotide sequence of the substrate containing dI. The PfuEndoQ cleavage site is indicated by an arrow. (**B**) 5′-Cy5-labeled substrates (10 nM) were incubated at 75°C for 15 min, as described in the Materials and Methods section. *I, 5′-Cy5-labeled 45-I25 ssDNA; *I/T 5′-Cy5-labeled 45-I25 annealed with 45R; I/*T, 5′-Cy5-labeled 45R annealed with 45-I25. M, 5′-Cy5-labeled ssDNA (23, 24, 25 and 26 nt) (lane 1); −, no enzyme (lanes 2, 4 and 6); +, 20-nM PfuEndoQ (lanes 3, 5 and 7). (**C**) 3′-FITC-labeled substrates (10 nM) were incubated. I*, 3′-FITC-labeled 45-I25 ssDNA; I*/T 3′-FITC-labeled 45-I25 annealed with 45R. M, 3′-FITC-labeled and 5′-phosphorylated 21-nt-ssDNA as a marker (lane 1); −, no enzyme (lanes 2 and 4); +, 20-nM PfuEndoQ (lanes 3 and 5). Cleavage products were separated by 8-M urea-12% PAGE.

The exact cleavage site of the detected endonuclease was investigated. Substrates with fluorescent labels at the 5′ or 3′ end of the dI-containing strand or the 5′-end of the complementary strand (Figure 4A) were cleaved by the purified PF1551 protein. As shown in Figure 4B (5′-Cy5-labeled substrates) and Figure 4C (3′-FITC-labeled substrates), the PF1551 protein cleaved the dI-containing DNA strand on the 5′-side of dI, and did not cleave the complementary strand. These results supported the identification of a novel endonuclease that nicks on the 5′-side of dI in the DNA strand, and is probably involved in the excision repair of dI in the archaeal cells. This novel enzyme was designated as endonuclease Q (EndoQ), due to its discovery at Kyu (Q) shu University.

### Recognition of dI-containing DNA

**Figure 5.**
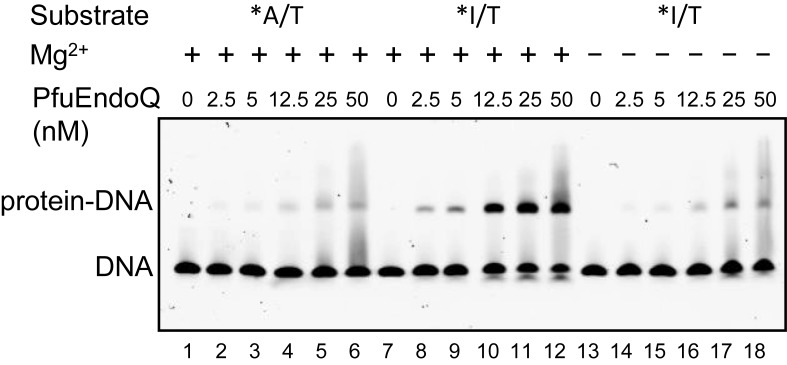
Electrophoresis mobility shift assay of PfuEndoQ. The dsDNA (5 nM) was incubated with various concentrations of PfuEndoQ (0, 2.5, 5, 12.5, 25 and 50 nM) in the presence (+) (lanes 1–12) or absence (−) (lanes 13–18) of 1-mM MgCl_2_. *A/T, 5′-Cy5-labeled dsDNA without dI (lanes 1–6); *I/T, 5′-Cy5-labeled dsDNA containing dI (lanes 7–18). The free DNA and the protein–DNA complex were separated by 4% native-PAGE.

The binding ability of PfuEndoQ to the dsDNA substrates was examined by using an electrophoresis mobility shift assay. PfuEndoQ showed specific binding to the dI-containing DNA (Figure 5). Stable complex formation was observed when an excess amount of PfuEndoQ was incubated with the dI-containing DNA, but not the normal duplex DNA, in the endonuclease reaction buffer in the presence of MgCl_2_. This observation indicated that PfuEndoQ remained bound to the nicked DNA product, as reported previously for PfuEndoV (21). To confirm the cleavage reaction, the samples from the gel shift experiments were subjected to denaturing gel electrophoresis. The results revealed that the shifted DNA in the gel shift assay was actually cleaved at the dI site (data not shown).

### Substrate specificity of PfuEndoQ

**Figure 6.**
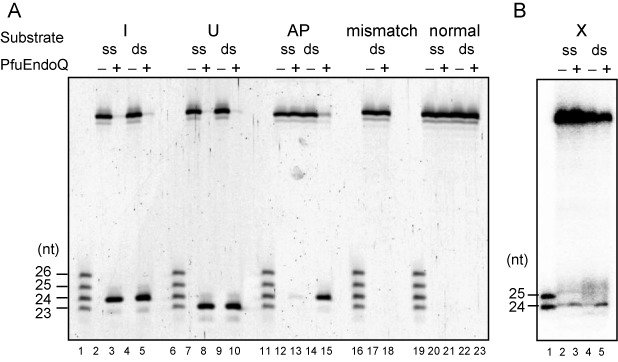
Substrate specificity of PfuEndoQ. (**A**) 5′-Cy5-labeled ssDNA (ss) or dsDNA (ds) substrates containing dI (lanes 2–5), dU (lanes 7–10), AP (lanes 12–15) and a mismatch (lanes 17 and 18) were subjected to reactions with PfuEndoQ. The normal DNAs (lanes 20–23) were used as the controls. The mixture of 5′-Cy5-labeled ssDNA (23, 24, 25 and 26 nt) was loaded on lanes 1, 6, 11, 16 and 19. The marker sizes are shown on the left of the panel. (**B**) 5′-^32^P-labeled ssDNA (ss) or dsDNA (ds) substrates containing dX (lanes 2–5) were subjected to the reactions with PfuEndoQ. The mixture of 5′-^32^P-labeled ssDNA (24 and 25 nt) was loaded on lane 1. The marker sizes are shown on the left of the panel.

To analyze the substrate specificity of the PfuEndoQ nuclease, we performed the cleavage reactions using substrates containing all three deaminated base lesions, a T/G mismatch base pair and an AP site. In addition to the clear endonuclease activity on the dI-containing dsDNA substrate, DNAs containing dU, dX and an AP site were also cleaved. In contrast, the normal ssDNA, dsDNA and the DNA containing a G/T mismatch were completely resistant to the cleavage reaction (Figure 6). The lengths of the products indicated that PfuEndoQ cleaved DNA on the 5′-side of the lesions. These results suggested that EndoQ is an endonuclease that functions in a repair process for damaged bases in archaeal cells.

### Cleavage activity and substrate specificity of TkoEndoQ

To investigate whether an EndoQ homolog from *T. kodakarensis* has similar properties, the gene encoding TK0887 was cloned and the recombinant protein was purified to homogeneity (Supplementary Figure S2A). TK0887 consists of 421 amino acid residues (MW 48 080.3). The cleavage assays of TK0887 were performed using the same substrate DNAs as in the case of PfuEndoQ. The cleaved fragments were observed when the substrates contained dI, dU, dX and an AP site, as observed for PfuEndoQ, and the cleaved sites were also the same (Supplementary Figure S2B and C). Hence, TK0887 is the functional homolog of PfuEndoQ, and the protein was designated as TkoEndoQ.

### Re-ligation of TkoEndoQ-cleaved products

**Figure 7.**
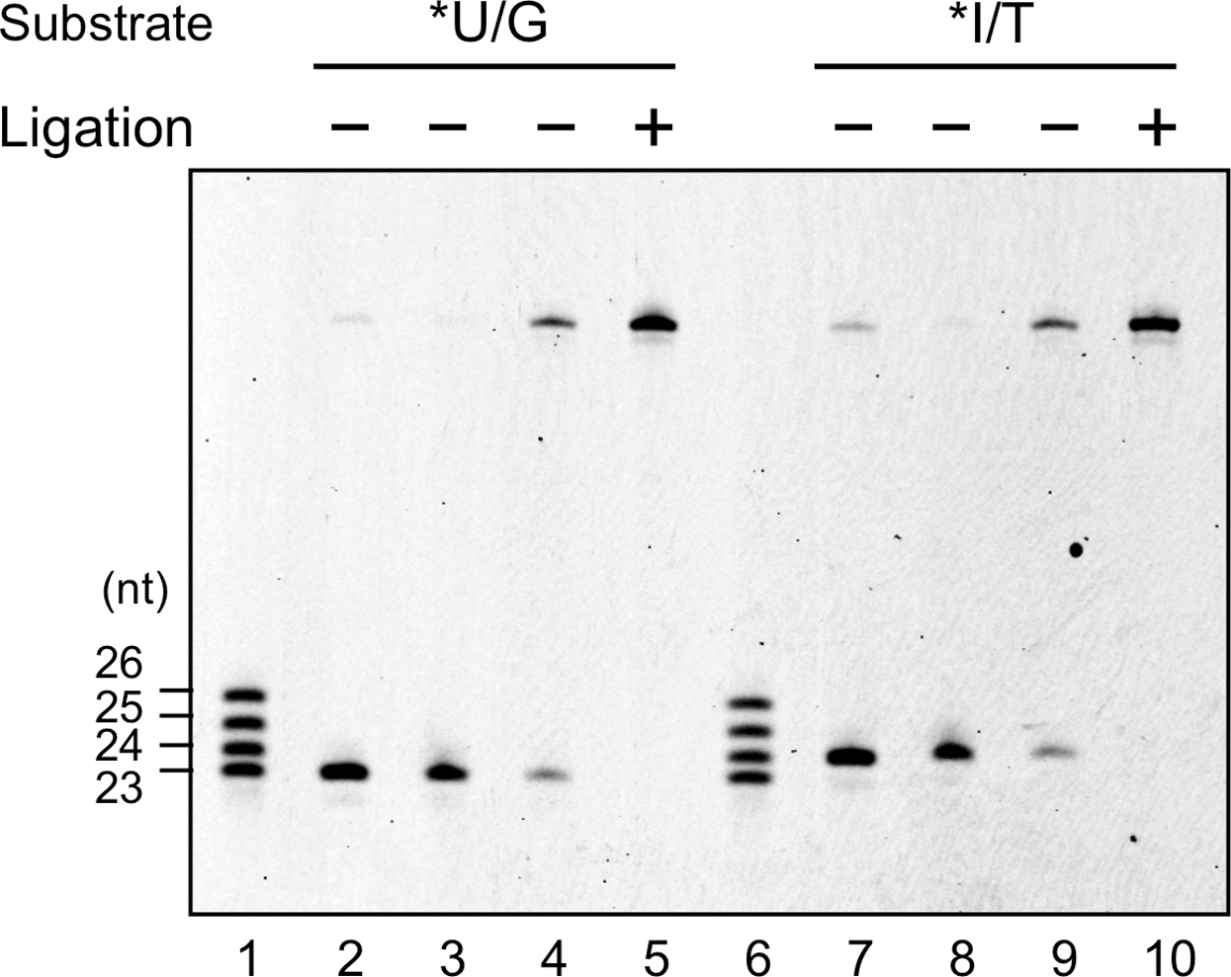
Re-ligation of the cleavage products from TkoEndoQ. *U/G, 5′-Cy5-labeled 45-U24 dsDNA (lanes 2–5). *I/T, 5′-Cy5-labeled 45-I25 dsDNA (lanes 7–10). Lanes 1 and 6, size marker (23, 24, 25 and 26 nt); lanes 2 and 7, cleaved products by TkoEndoQ (negative controls); lanes 3 and 8, annealed DNA without ligation; lanes 4 and 9, ligated products; lanes 5 and 10, intact substrates (positive controls).

To investigate whether EndoQ gives rise to ligatable products, re-ligation of the cleaved products of TkoEndoQ was performed. As shown in Figure 7, TkoEndoQ produced a visible DNA fragment (5′-labeled) that was either 23- or 24-nt long (lanes 2 and 7). After the cleavage products were denatured, these DNAs were re-annealed and treated with T4 DNA ligase. The reaction converted the 23- or 24-nt-long DNA to the full length of each substrate (45 nt) (lanes 4 and 9). The lengths of the cleavage products did not change without the T4 DNA ligase treatment (lanes 3 and 8). Thus, TkoEndoQ leaves a ligatable nick (5′-phosphate and 3′-hydroxyl termini) on substrates after the cleavage, and this property is suitable for the quick repair process.

### Detection and estimation of the number of EndoQ molecules in *T. kodakarensis* cells

**Figure 8.**
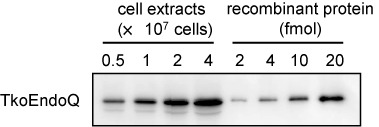
Estimation of the amount of TkoEndoQ in *T. kodakarensis* cells. The *T. kodakarensis* cells at exponential phases were harvested, and the whole cell extracts from the indicated numbers of the cells were subjected to western blot analysis. To create a standard curve, the serially diluted recombinant TkoEndoQ protein was subjected to the western blot analysis in parallel, and the band intensities were quantified by an image analyzer.

To confirm that EndoQ is produced in *Thermococcal* cells, a western blotting analysis was performed. Using the anti-TkoEndoQ antiserum, a major band with the same size as the recombinant protein was detected from the total extract of *T. kodakarensis* cells (Figure 8). A standard curve was created by the band intensities of the serially diluted recombinant TkoEndoQ protein, and the number of TkoEndoQ molecules in *T. kodakarensis* was estimated to be 1 × 10^3^ per cell.

### Amino acid sequence analysis and phylogenetic analysis of EndoQ

**Figure 9.**
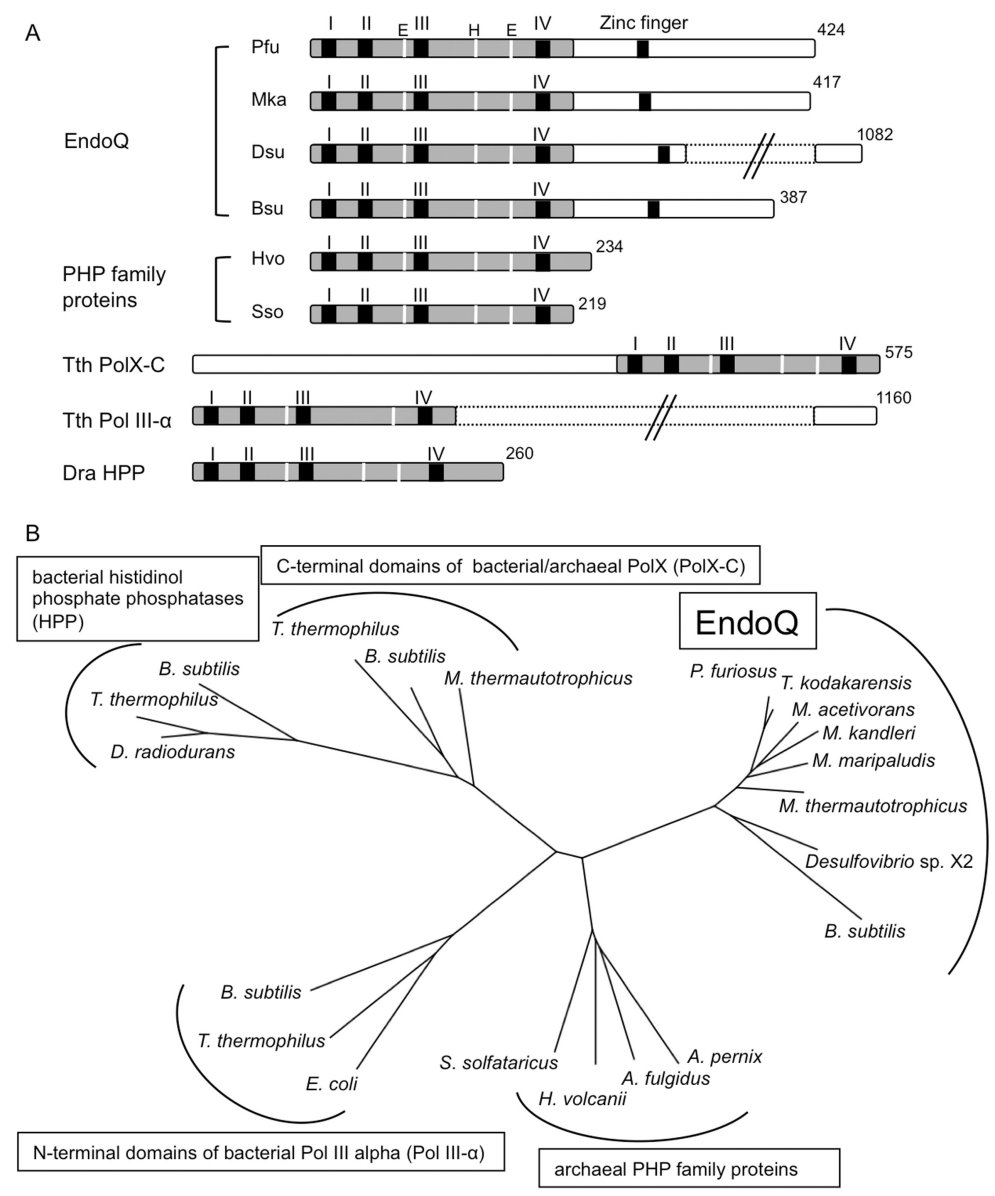
Schematic drawing of the domain structure, and an unrooted phylogenetic tree of the EndoQ proteins. (**A**) The domain structures of the EndoQ proteins from Archaea and Bacteria are shown, with their total amino acid numbers. The conserved PHP domains (shaded in gray) and zinc finger motifs (black bar) are indicated in each protein. The highly conserved motifs I–IV (black bars), Glu and His (white lines) are indicated in the PHP domains of each protein. (**B**) An unrooted phylogenetic tree of PHP domains showing five clusters. Twenty one sequences were retrieved from the protein database at NCBI. The sequences were aligned by MAFFT (http://mafft.cbrc.jp/alignment/software/) and were further adjusted by hand. The alignment was used in generating a phylogenetic tree based on the neighbor-joining method. For a detailed description and the full names of organisms and accession numbers of proteins, see Supplementary Figure S3.

The deduced amino acid sequence of EndoQ is clearly conserved in *Thermococcals* and some of the methanogens in Archaea. No known protein from Eukarya in the public database is similar to EndoQ. However, it is interesting that some bacteria, including *Bacillus* and *Desulfovibrio* genus, have a homolog (Figure 9A). EndoQ has a PHP domain-like sequence in the N-terminal region, and can be included in a phylogenetic tree of PHP domain-containing proteins from Archaea and Bacteria (Figure 9B). EndoQ seems to have originated from the ancestor of archaea and evolved in the archaeal domain, specifically in *Thermococcals* and some methanogens.

## DISCUSSION

In this study, we identified a novel endonuclease, PF1551 and TK0887 from *P. furiosus* and *T. kodokarensis*, respectively, that cleaves the phosphodiester bond on the 5′-side of a nucleotide with a damaged base. The activity to cleave the closest phosphodiester bond 5′ from the damaged base is not common in the known BER process. Furthermore, the amino acid sequences of the corresponding proteins are not homologous to any known enzymes. Therefore, we designated this novel nuclease as Endonuclease Q (EndoQ). This discovery provides great insights into the identification of the novel repair pathway of damaged DNA strands in organisms that live in an extreme environment and also into the evolution of repair enzymes.

### EndoQ homologs in archaea

A sequence comparison of PfuEndoQ and TkoEndoQ with other proteins in the databases revealed some related proteins (Figure 9A). EndoQ has a PHP domain, which includes conserved motifs with other proteins, such as some family C and X DNA polymerases (22). EndoQ contains four cysteines in the C-terminal region (Supplementary Figure S3). This region might be a specific signature of the EndoQ protein family. These cysteines may be an iron–sulfur cluster, which is observed in many DNA replication and repair enzymes. However iron–sulfur proteins are generally yellow-colored. The solutions of purified PfuEndoQ and TkoEndoQ are colorless even at 50 μM. Therefore, this cysteine cluster is predicted to be a zinc-binding site with consensus sequence of CPXCG (23). This zinc finger could be possibly involved in the interaction with DNA, RNA or nucleic acid-related proteins. A cluster consisting of the archaeal PHP family proteins in the phylogenetic tree (Figure 9B) includes proteins with only the PHP domain (Figure 9A). These proteins in this cluster may lack the EndoQ activity. To investigate the functions of the C-terminal region of EndoQ, we are characterizing the protein from *Sulfolobus tokodaii*, in addition to creating mutants of TkoEndoQ with deletions in the C-terminal region. Moreover, many archaea, including *Thermoproteales* (except for *Thermofilum pendens*), *Thermoplasmatales* and *Methanocellales* and also those in the phyla of Korarchaeota, Nanoarchaeota, Thaumarchaeota and Aigarchaeota, do not have any protein with homology to even the N-terminal PHP of EndoQ. Furthermore, it is also noteworthy that some bacteria have the EndoQ homolog. These bacterial homologs share the sequence similarity along the entire region with EndoQ, and may have a similar damaged base-specific endonuclease activity. We have started to study the bacterial EndoQ using the *B. subtilis* homolog, to understand the meaning of this distribution of EndoQ. Our preliminary experiment showed that the *B. subtilis* homolog actually has the EndoQ-like activity. The gene disruption analyses using *B. subtilis* will provide important information about whether the retention of the *endoQ* gene was needed in bacterial cells during evolution.

Archaeal and bacterial genomes sometimes contain foreign genes coming from a still largely unexplored reservoir of integrative elements. Survey of potential recently acquired integrative elements in 119 archaeal and bacterial genomes has been reported (24). EndoQ has not been discovered in the integrated mobile elements (24,25). From these observations, the origin of EndoQ is mysterious. We constructed a phylogenetic tree with only EndoQ proteins to see if they have co-evolved with Archaea and where would be the location of the bacterial members (Supplementary Figure S4). The topology of clusters was similar to the evolutionary relationship obtained by 16S rDNA. Therefore, EndoQ seems to have come into existence during evolution in the archaeal domain, and may serve as an enzyme for the specific repair of damaged bases in some archaeal organisms. It is not clear why only a few bacterial genuses have a gene encoding an EndoQ homolog.

### Substrate recognition and specificity of PfuEndoQ

The cleavage reaction by EndoQ produced a 5′-phosphate and a 3′-OH at the incised site, which is common to all of the metal-ion-dependent nuclease reactions (26). Actually, EndoQ required a divalent cation to recognize the damaged base, because the binding of EndoQ to dI-containing DNA is Mg^2+^-dependent (Figure 5). Several highly conserved residues, such as D6, L7, H8, H10, H139, S191, D192 A193 and H194, are present in the PHP domains of PfuEndoQ (Supplementary Figure S3). In addition to the most conserved ‘SDAH’ motif, several more residues, such as E76, H84 and E167, may also be important for the EndoQ function. Mutational analyses of these residues, as well as the crystallographic analyses of PfuEndoQ itself and in its complex with damaged base-containing DNA, are now in progress to understand the structure and functions of this new endonuclease. These studies will facilitate the elucidation of the molecular mechanism of the EndoQ reaction.

### EndoQ-mediated repair pathways

**Figure 10.**
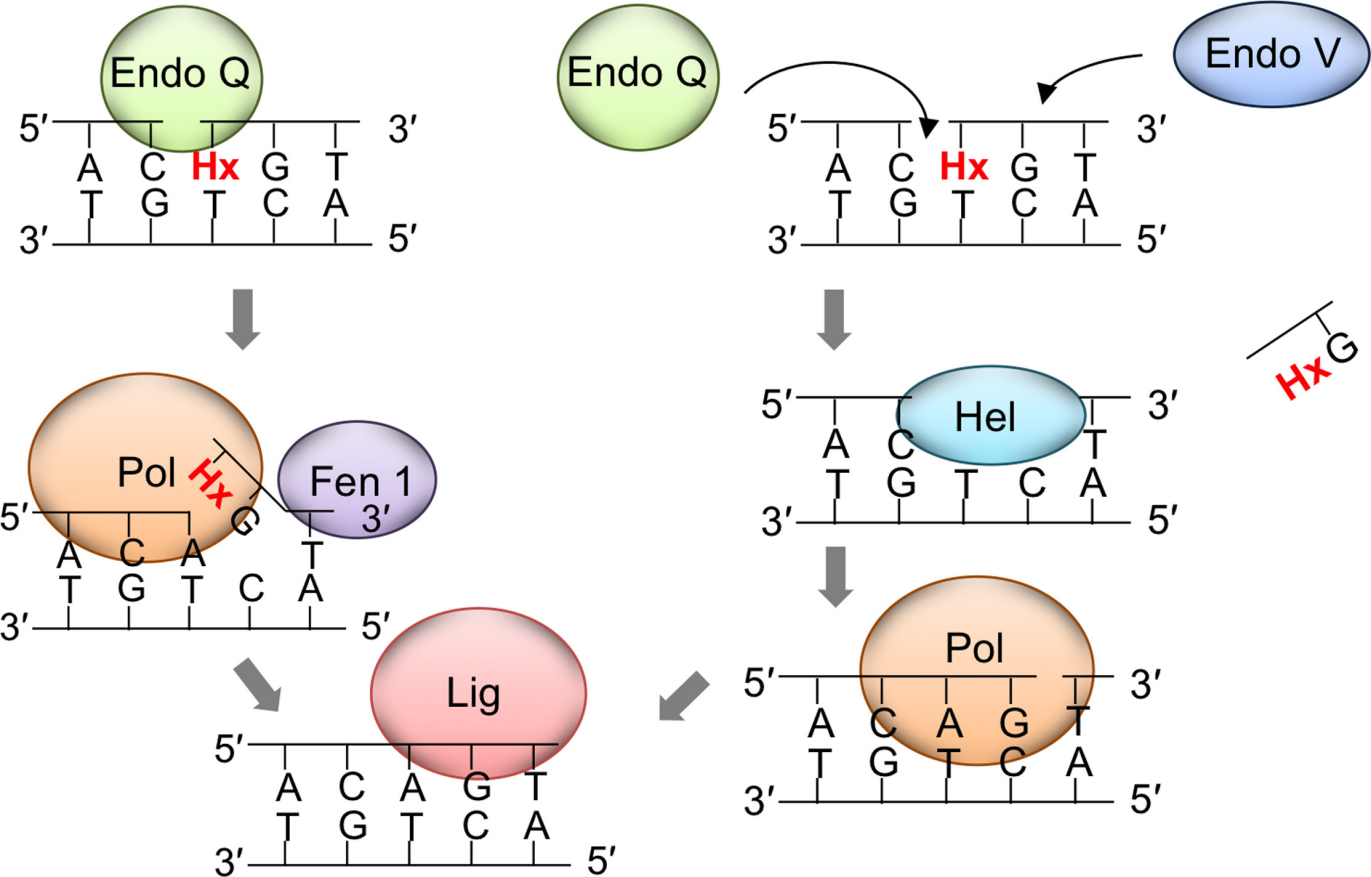
Models for the EndoQ-mediated dI repair pathway. Left pathway: EndoQ recognizes dI in DNA and incises on its 5′-side. A DNA polymerase is recruited to the nicked site and synthesizes a new DNA strand coupled with the 5′–3′ strand displacement of the forward strand, and then Fen1 cuts off the resulting flap before DNA ligase seals the nick. Right pathway: EndoQ and EndoV cooperate in excising the damaged DNA portion, by cleaving on its 5′- and 3′-sides, respectively, followed by removal by helicase activity. Subsequently, a DNA polymerase fills the gap and DNA ligase seals the nick.

We consider two possible pathways including EndoQ for the repair of DNA containing a damaged base. First, EndoQ recognizes the damaged base in DNA and hydrolyzes the bond on its 5′-side, to nick the DNA strand. A DNA polymerase (Pol) is recruited to the nicked site and synthesizes a new DNA strand from the 3′-terminus with displacement of the downstream strand. Then, Flap-endonuclease 1 (Fen1) cuts off the resulting flap, and the resultant nick is connected by DNA ligase (Lig) (Figure 10, left pathway). Alternatively, a 5′–3′ exonuclease may remove the dI from DNA (26). This may be a relatively shorter version of the conventional BER using UDG and AP endonuclease (APE). A similar pathway has been reported, in which an ExoIII homolog, Mth212, from *Methanothermobacter thermoautotrophicus* functions as a uridine endonuclease cleaving on the 5′-side of dU (27). The amino acid sequence of Mth212 shares 30% identity with *E. coli* ExoIII and 40% identity with human ApeI (28). It would be very interesting to investigate the differences between the ExoIII homologs with or without the uridine endonuclease activity. No gene encoding a UDG homolog was found in the *M. thermoautotrophicus* genome, and no UDG activity has been detected in the cell extract of this strain (27). A reasonable speculation is that the direct cleavage activity on the 5′-side of dU was acquired, instead of the two-step procedure by the UDG-APE reactions. This ExoIII-dependent uridine repair procedure was biochemically demonstrated, and this repair process probably functions in the methanogen (29). No ExoIII homolog has been found in the *P. furiosus* and *T. kodakarensis* genomes (Supplementary Table S3). Instead, EndoQ may be responsible for this one-step procedure, at the beginning of archaeal BER. However, these *Thermococcals* have UDG and APE to cleave the glycoside bond of dU and the phosphodiester bond of the subsequent abasic site, respectively (30,31). The EndoQ-dependent and UDG-APE-dependent processes may complement each other for a more efficient BER system in these hyperthermophiles.

The EndoQ-dependent process may be a type of AER, initiated by a single nick near the damaged site. AER is an excision repair system for UV damage, and the UV damage endonuclease (UVDE) is the enzyme responsible for introducing a nick immediately 5′ to the various types of UV lesions, as well as other types of base damage (5). The distribution of UVDE in the genome database revealed that most of the archaeal genomes, including *Pyrococcus* and *Thermococcus*, do not have a gene encoding a homologous sequence to UVDE (32). Therefore, we investigated whether EndoQ has cleavage activity for DNA substrates including a cyclobutane pyrimidine dimer (CPD) or (6-4) photoproducts (6-4 PPs). *In vitro* enzyme assays under the same conditions as those for the DNAs containing deaminated bases showed that neither CPD- nor 6-4 PPs-containing DNA was cleaved by PfuEndoQ (Supplementary Figure S5). Therefore, EndoQ does not seem to be involved in UV-damage repair, which may not be required for *Pyrococcus* living in the deep sea environment.

As an alternative pathway, EndoQ and EndoV may act in succession in the repair process (Figure 10, right pathway). EndoV hydrolyzes the second phosphodiester bond on the 3′-side from the damaged base, and EndoQ cleaves on its 5′-side. These simultaneous or successive hydrolysis reactions cut off the DNA portion containing the damaged base, and the resultant gap is filled by Pol and Lig. This hypothesis is based on the fact that EndoV remains bound to dI-containing DNA, even after cleaving the phosphodiester bond. This feature suggests that EndoV may serve as a recruiter of other enzymes, as predicted previously (21). It is not presently certain whether EndoV and EndoQ interact with each other, and further experiments investigating protein–protein interactions are now underway. The *in vitro* reconstitution of the repair process including EndoQ and EndoV will also be an important subject. Recent reports showed that the human EndoV (hEndoV) prefers RNA rather than DNA as its substrate, and suggested that it is involved in RNA metabolism in human cells (33,34). Our analyses of the *P. furiosus* EndoV revealed that it also cleaves an inosine-containing RNA strand efficiently (21). Currently, it is not known whether the archaeal EndoV functions in the metabolism of DNA, RNA or both in archaeal cells. Genetic analyses using the *endoV* mutant strain are necessary to obtain a more definitive answer for this question. We analyzed whether EndoQ cleaves the damaged base-containing RNA, as well as DNA. However, EndoQ never cleaved either double-stranded or single-stranded RNA, under the same conditions as the reactions for DNA, in contrast to the case of EndoV (Supplementary Figure S6). Therefore, EndoQ certainly functions as a DNA repair enzyme in the cells. A genetic manipulation system is now available for *T. kodakarensis* (35,36), and the isolation of gene knock-out mutants for EndoQ, EndoV, UDG and APE from the *T. kodakarensis* genome is now in progress. The DNA repair systems in the hyperthermophilic archaea are poorly understood to date. These studies will advance our knowledge of the DNA repair systems of Extremophiles.

## SUPPLEMENTARY DATA

Supplementary Data are available at NAR Online.

SUPPLEMENTARY DATA
