# Supplementary material for: A novel endonuclease that may be responsible for damaged DNA base repair in Pyrococcus furiosus
Source: Nucleic Acids Res. 2015 Feb 18;43(5):2853–63. doi: 10.1093/nar/gkv121 (PMC4357722; doi:10.1093/nar/gkv121)
Supplement: SUPPLEMENTARY DATA [file supp_43_5_2853__index.html]

A novel endonuclease that may be responsible for damaged DNA base repair in Pyrococcus furiosus — SUPPLEMENTARY DATA 

# A novel endonuclease that may be responsible for damaged DNA base repair in *Pyrococcus furiosus*

## SUPPLEMENTARY DATA

**Files in this Data Supplement:**

- Supplemental Information
