# Supplementary material for: A novel endonuclease that may be responsible for damaged DNA base repair in Pyrococcus furiosus
Source: Nucleic Acids Res. 2015 Feb 18;43(5):2853–63. doi: 10.1093/nar/gkv121 (PMC4357722; doi:10.1093/nar/gkv121)
Supplement: SUPPLEMENTARY DATA [file supp_gkv121_nar-03740-d-2014-File004.pdf]

Table S1 | DNA substrates

Substrate name	(nt)	Nucleotide sequence
49N	49	5′-dAGCTACCATGCCTGCACGAATTAAGCAATTCGTAATCATGGTCATAGCT-3′
49-I25	49	5′-dAGCTACCATGCCTGCACGAATTAA**I**CAATTCGTAATCATGGTCATAGCT-3′
49-X25	49	5′-dAGCTACCATGCCTGCACGAATTAA**X**CAATTCGTAATCATGGTCATAGCT-3′
49R	49	5′-dAGCTATGACCATGATTACGAATTGCTTAATTCGTGCAGGCATGGTAGCT-3′
45N	45	5′-dCGAACTGCCTGGAATCCTGACGACATGTAGCGAACGATCACCTCA-3′
45-I25	45	5′-dCGAACTGCCTGGAATCCTGACGAC**I**TGTAGCGAACGATCACCTCA-3′
45-U24	45	5′-dCGAACTGCCTGGAATCCTGACGA**U**ATGTAGCGAACGATCACCTCA-3′
45-AP25	45	5′-dCGAACTGCCTGGAATCCTGACGAC**(AP)**TGTAGCGAACGATCACCTCA-3′
45-G25	45	5′-dCGAACTGCCTGGAATCCTGACGAC**G**TGTAGCGAACGATCACCTCA-3′
45R	45	5′-dTGAGGTGATCGTTCGCTACATGTCGTCAGGATTCCAGGCAGTTCG-3′
49- dsDNA		Prepared by annealing of 49 (N, -I25, X25) and complementary 49R
45- dsDNA		Prepared by annealing of 45 (N, -I25, U24, AP25, G25) and complementary 45R

Table S2| Identification of proteins by MS snalysis.

slice*	locus tag	annotation by NCBI
1	PF0053	ATP dependent RNA helicase
	PF1717	translation initiation factor eIF-2, subunit gamma
	PF1123	hypothetical protein
	PF1615	hypothetical protein
2	PF0041	oxidative cyclase
	PF1717	translation initiation factor eIF-2, subunit gamma
3	PF1615	hypothetical protein
	PF0301	cobyric acid synthase
	PF1717	translation initiation factor eIF-2, subunit gamma
	PF1123	hypothetical protein
	PF1140	translation initiation factor eIF-2, subunit alpha
4	PF1123	hypothetical protein
	PF1717	translation initiation factor eIF-2, subunit gamma
	PF1615	hypothetical protein
	PF1551	hypothetical protein
	PF0124	hypothetical protein
5	PF1717	translation initiation factor eIF-2, subunit gamma
	PF1123	hypothetical protein
	PF1551	hypothetical protein
	PF0124	hypothetical protein
	PF0251	hypothetical protein
6	PF0124	hypothetical protein
	PF1717	translation initiation factor eIF-2, subunit gamma
	PF1257	nol1-nop2-sun family nucleoar protein
	PF1414	annotated as flap endonuclease 1 (rad2)
7	PF1140	translation initiation factor eIF-2, subunit alpha
	PF0875	hypothetical protein
	PF0258	endonuclease IV
	PF0279	ABC transporter
8	PF1862	DNA-binding protein
	PF1385	DNA polymerase, bacteriophage-type
	PF0988	hypothetical protein
	PF1414	flap structure-specific endonuclease
	PF1776	hypothetical protein

* The proteins in fraction 48 of the MonoS column were separated by SDS-10 % PAGE and the gel slices were analyzed by MS. 16

Table S3 Conservation of the candidate genes for the repair of Uracil-containing DNA

	EndoQ	Uracil-DNA glycosylase				Endonuclease IV		Exonuclease III	
Organism	arCOG04881	arCOG00905				arCOG01894		arCOG02207	
Euryarchaeota									
Thermococcales									
Pyrococcus abyssi	PAB0431	PAB0474				PAB1103			
Pyrococcus furiosus	PF1551	PF1385				PF0258			
Pyrococcus horikoshii	PH1535	PH1472				PH1905			
Thermococcus gammatolerans	TGAM_0434	TGAM_1814	TGAM_0543			TGAM_1446		TGAM_0365	
Thermococcus kodakaraensis	TK0887	TK2143				TK0170			
Thermococcus onnurineus	TON_0421	TON_1518	TON_0680			TON_1961			
Thermococcus sibiricus	TSIB_0578	TSIB_0879	TSIB_1579			TSIB_0912		TSIB_0948	
Halobacteriales									
Haloarcula marismortui		rrnAC0123	rrnAC2636	rrnAC2796	rrnAC0005	rrnAC2015			
Halobacterium salinarum R1		OE2052F	OE2758R	OE3906F		OE1304F			
Halobacterium sp		VNG0707C	VNG1228C	VNG2082G		VNG0183G			
Halomicrobium mukohataei DSM 12286		Hmuk_0717	Hmuk_2639	Hmuk_2571		Hmuk_0185			
Haloquadratum walsbyi		HQ1380A	HQ2967A			HQ1417A			
Halorhabdus utahensis DSM 12940		Huta_0183	Huta_1768	Huta_2974		Huta_2402			
Halorubrum lacusprofundi ATCC 49239		Hlac_0379	Hlac_0610	Hlac_1992		Hlac_0531			
Natronomonas pharaonis		NP0084A	NP3952A	NP5088A		NP5118A			
Methanococcales									
Methanocaldococcus fervens	Mefer_0424					Mefer_1144	Mefer_1161		
Methanocaldococcus jannaschii	MJ0043					MJ0133			
Methanocaldococcus vulcanius M7	Metvu_0306					Metvu_1373	Metvu_1445		
Methanococcus aeolicus	Maeo_1319					Maeo_1039			
Methanococcus maripaludis S2	MMP0095					MMP1678		MMP1012	
Methanococcus maripaludis C5	MmarC5_1581					MmarC5_1729		MmarC5_0582	
Methanococcus maripaludis C6	MmarC6_0852					MmarC6_0994		MmarC6_1647	
Methanococcus maripaludis C7	MmarC7_1094					MmarC7_0951		MmarC7_0254	
Methanococcus vannielii SB	Mevan_1107					Mevan_0978		Mevan_0339	
Mathanomicrobiales									
Methanoregula boonei	Mboo_1948					Mboo_0654		Mboo_0350	Mboo_2415
Methanosphaerula palustris E1 9c	Mpal_0960					Mpal_1968			
Methanocorpusculum labreanum Z	Mlab_1296	Mlab_1146				Mlab_0226			
Methanoculleus marisnigri JR1		Memar_2334				Memar_0548	Memar_0638	Memar_1440	
Methanospirillum hungatei	Mhun_1125					Mhun_1782		Mhun_0441	
Methanosarcinales									
Methanococcoides burtonii	Mbur_1399	Mbur_2338				Mbur_2015		Mbur_2145	
Methanosarcina acetivorans	MA0641	MA3593	MA2265			MA3548		MA2077	
Methanosarcina barkeri	Mbar_A1564	Mbar_A1809				Mbar_A2341		Mbar_A3242	
Methanosarcina mazei	MM_1805	MM_0486				MM_0460		MM_3148	
Methanosaeta thermophila	Mthe_0764	Mthe_1479				Mthe_0406			

Thermoplasmatales										
Picrophilus torridus		PTO0989	PTO0919	PTO1020		PTO0616		PTO0627		
Thermoplasma acidophilum		Ta0477				Ta0096	Ta0891	Ta1506m		
Thermoplasma volcanium		TVN0827				TVN0157	TVN0971	TVN0046		
Methanobacteriales										
Methanothermobacter thermoautotrophicus	MTH1305					MTH1010		MTH212		
Methanosphaera stadtmanae	Msp_1523					Msp_0992				
Methanobrevibacter smithii						Msm_0963		Msm_1479		
Methanopyrales										
Methanopyrus kandleri	MK0424									
Archaeoglobales										
Archaeoglobus fulgidus		AF2277						AF0580		
environmental samples										
Uncultured methanogenic archaeon RC-I		RRC526				RRC305		RCIX1223	RCIX2491	RCIX2501
Crenarchaeota										
Sulfolobales										
Sulfolobus acidocaldarius		Saci_0159	Saci_1756			Saci_0015		Saci_0129		
Sulfolobus islandicus L S 2 15		LS215_0085	LS215_2750			LS215_2937		LS215_0101		
Sulfolobus islandicus M 14 25		M1425_0085	M1425_2591			M1425_2772		M1425_0101		
Sulfolobus islandicus M 16 27		M1627_0085	M1627_2644			M1627_2824		M1627_0101		
Sulfolobus islandicus M 16 4		M164_0085	M164_2575			M164_2753		M164_0101		
Sulfolobus islandicus Y G 57 14		YG5714_0087	YG5714_2757			YG5714_2952		YG5714_0103		
Sulfolobus islandicus Y N 15 51		YN1551_0085	YN1551_0133			YN1551_3142		YN1551_0101		
Sulfolobus solfataricus		SSO2275	SSO2733			SSO2156		SSO2290		
Sulfolobus tokodaii		ST2238	ST2405			ST2148		ST1910		
Metallosphaera sedula		Msed_0720	Msed_2071			Msed_0729		Msed_1434		
Thermoproteales										
Thermoproteus neutrophilus V24Sta		Tneu_1169	Tneu_1948			Tneu_1593				
Caldivirga maquilingensis		Cmaq_1649	Cmaq_0814			Cmaq_0997		Cmaq_1739		
Pyrobaculum aerophilum		PAE0651	PAE1327			PAE3257				
Pyrobaculum arsenaticum		Pars_2305				Pars_1689				
Pyrobaculum calidifontis		Pcal_2076				Pcal_1781				
Pyrobaculum islandicum		Pisl_0718	Pisl_1921			Pisl_0510				
Thermofilum pendens		Tpen_0798				Tpen_0883				
Desulfurococcales										
Staphylothermus marinus F1		Smar_1316				Smar_1185				
Desulfurococcus kamchatkensis		DKAM_0281				DKAM_0986				
Ignicoccus hospitalis		Igni_0301				Igni_1092				
Aeropyrum pernix		APE_0427.1				APE_2104.1				
Hyperthermus butylicus		Hbut_0091				Hbut_1039				
Cenarchaeales										
Cenarchaeum symbiosum A		CENSYa_059[illegible]	CENSYa_1913			CENSYa_1925				
Korarchaeota										
Korarchaeum cryptofilum OPF8		Kcr_0977				Kcr_0075				

Thaumarchaeota			
Nitrosopumilus maritimus SCM1	Nmar_0366	Nmar_1056	Nmar_0064
Aigarchaeota			
Caldiarchaeum subterraneum	CSUB_C1151		CSUB_C1167
Nanoarchaeota			
Nanoarchaeum equitans	NEQ372		

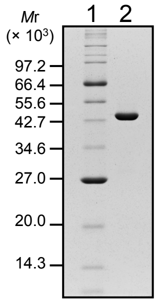

Figure S1. **Preparation of recombinant PF1551.** The protein marker (lane 1) and the purified PF1551 (1 μg) (lane 2) was subjected to SDS-12% PAGE followed by CBB staining. The sizes of markers are shown on the left of the panel.

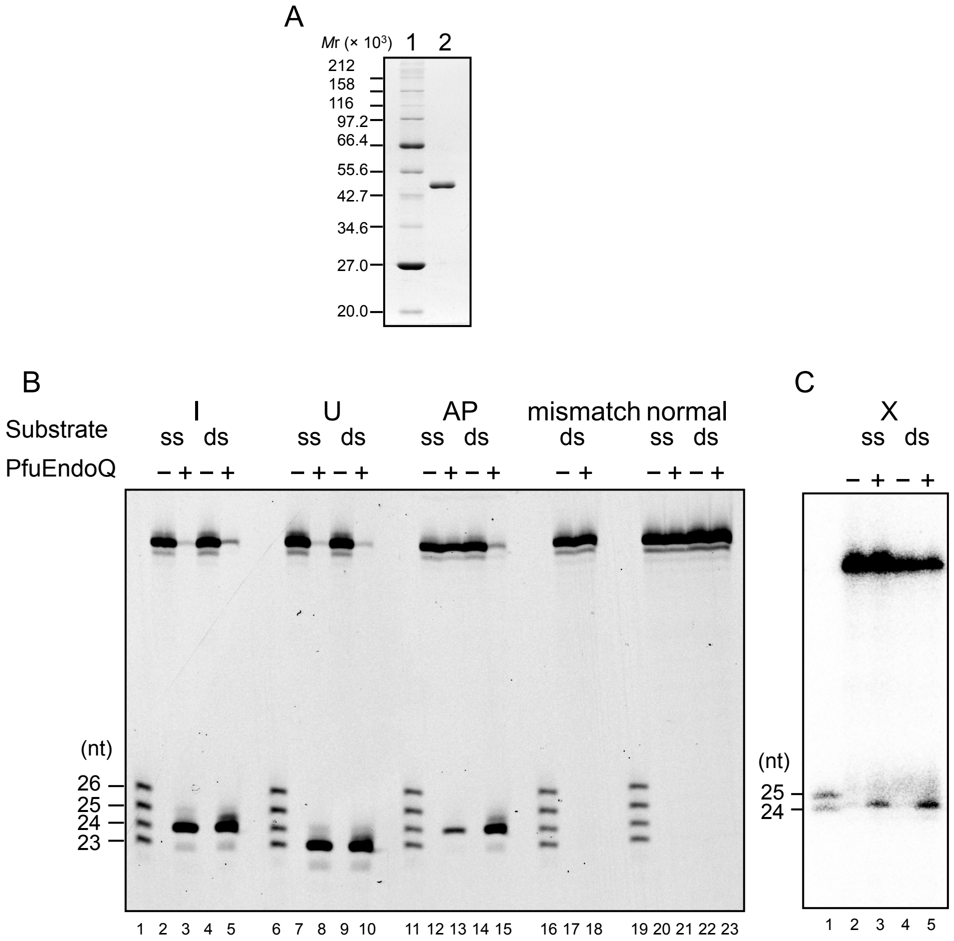

Figure S2. **Properties of the recombinant TK0887 protein.** (A) The protein markers (lane 1) and the purified TK0887 protein (1 µg) (lane 2) were subjected to SDS-12% PAGE followed by CBB staining. The marker sizes are shown on the left of the panel. (B) Substrate specificity of TkoEndoQ. 5′-Cy5-labeled ssDNA (ss) or dsDNA (ds) substrates containing dI (lanes 2-5), dU (lanes 7-10), AP (lanes 12-15), and a mismatch (lanes 17 and 18) were subjected to the reactions with TkoEndoQ. The normal DNA (lanes 20-23) was used as the controls. The mixture of 5′-Cy5-labeled ssDNA (23, 24, 25, and 26 nt) was loaded on lanes 1, 6, 11, 16, and 19. The marker sizes are shown on the left of the panel. (C) 5′-32P-labeled ssDNA (ss) or dsDNA (ds) substrates containing dX (lanes 2-5) were subjected to the reactions with TkoEndoQ. The mixture of 5′-32P-labeled ssDNA (24 and 25 nt) was loaded in lane 1. The marker sizes are shown on the left of the panel.

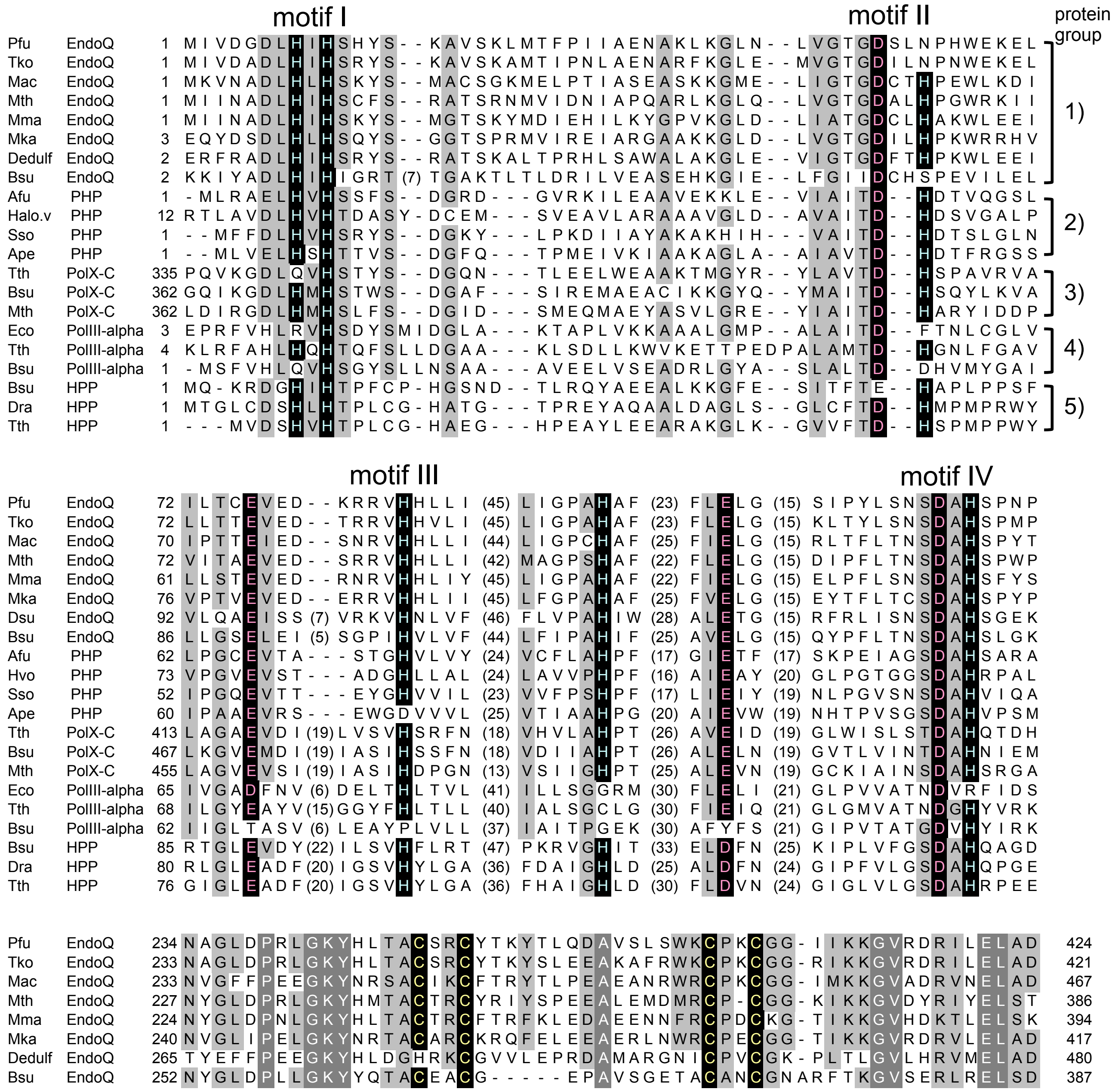

***Figure S3.* Alignment of the PHP domains and the C-terminal region of EndoQ homologs.** The protein group are 1) EndoQ, EndoQ from *P. furiosus*, *T. kodakarensis*, and homologous proteins from archaea and bacteria; 2) PHP, proteins including PHP domain but lacking a detectable zinc finger motif, from archaea; 3) PolX-C, C-terminal domains of family X DNA polymerases from bacteria and archaea; 4) PolIII-alpha, N-terminal domains of the bacterial DNA polymerase III α subunits; 5) HPP, bacterial histidinol phosphate phosphatases. The species abbreviations (accession numbers in parentheses) are: group 1), Pfu, *P. furiosus* (NP_579280); Tko, *T. kodakarensis* (YP_183300); Mac, *Methanosarcina acetivorans* C2A (NP_615603); Mth, *Methanothermobacter thermautotrophicus* str. ΔH (NP_615603); Mma, *Methanococcus maripaludis* C6 (YP_001548901); Mka, *Methanopyrus kandleri* AV19 (NP_613709); Dsu, *Desulfovibrio* sp. X2 (WP_020880034); Bsu, *Bacillus subtilis* (WP_003236760); group 2), Afu, *Archaeoglobus fulgidus* DSM 4304 (NP_069341); Hvo, *Haloferax volcanii* DS2 (YP_003535996); Sso, *Sulfolobus solfataricus* P2 (NP_342371); Ape, *Aeropyrum pernix* K1 (NP_146951); group 3), Tth, *Thermus thermophilus* HB8 (YP_144416); Bsu, *B. subtilis* str. 168 (NP_390737); Mth, *M. thermautotrophicus* str. Delta H (NP_275693); group 4), Eco, *Escherichia coli* (DPO3A_ECOLI); Tth *T. thermophilus* HB8 (YP_143446); *B. subtilis* subsp. str. 168 (NP_390801); group 5), *B. subtilis* str. 168 (NP_390840); Dra, *Deinococcus radiodurans* R1 (NP_294193); Tth, *T. thermophilus* HB8 (YP_143597). Alignment was performed with MAFFT (http://mafft.cbrc.jp/alignment/software/). Motifs I–IV, present in the PHP domain, correspond to the boxed areas. The highly conserved histidine, glutamate and aspartate residues proposed to act as metal ligands at the PHP domain are indicated in cyan (histidines) and magenta (glutamates and aspartates) letters, over a black background. Other highly conserved (over 80% similarity) residues are shown over a gray background. Cysteines in the Zinc finger motif are indicated in yellow over a black background. Other highly conserved residues are indicated in white over a dark gray background (100% identity). Numbers on the left and in the parenthesis indicate the amino acid position relative to the N-terminus, and omitted residues, respectively.

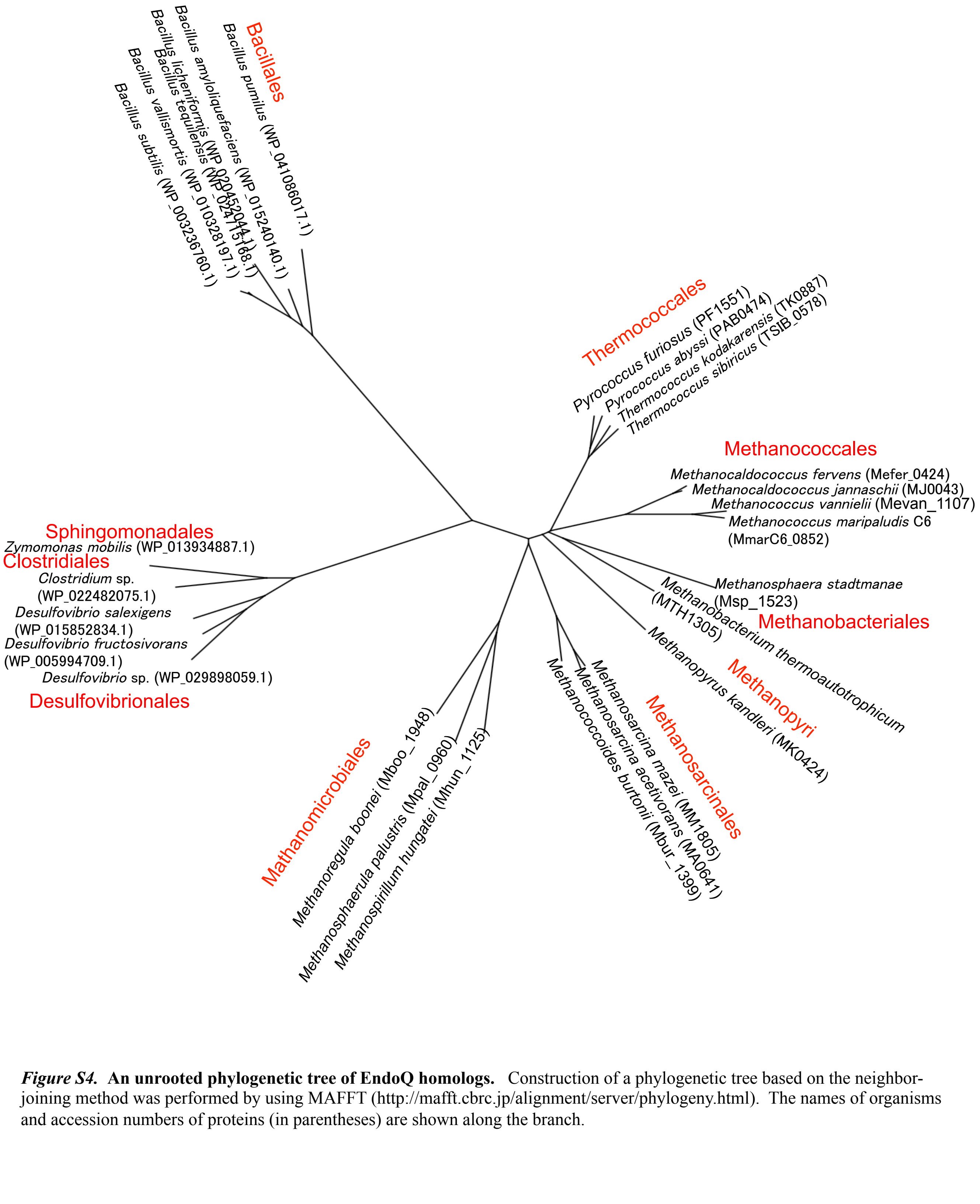

Figure S4. **An unrooted phylogenetic tree of EndoQ homologs.** Construction of a phylogenetic tree based on the neighbor-joining method was performed by using MAFFT (http://mafft.cbrc.jp/alignment/server/phylogeny.html). The names of organisms and accession numbers of proteins (in parentheses) are shown along the branch.

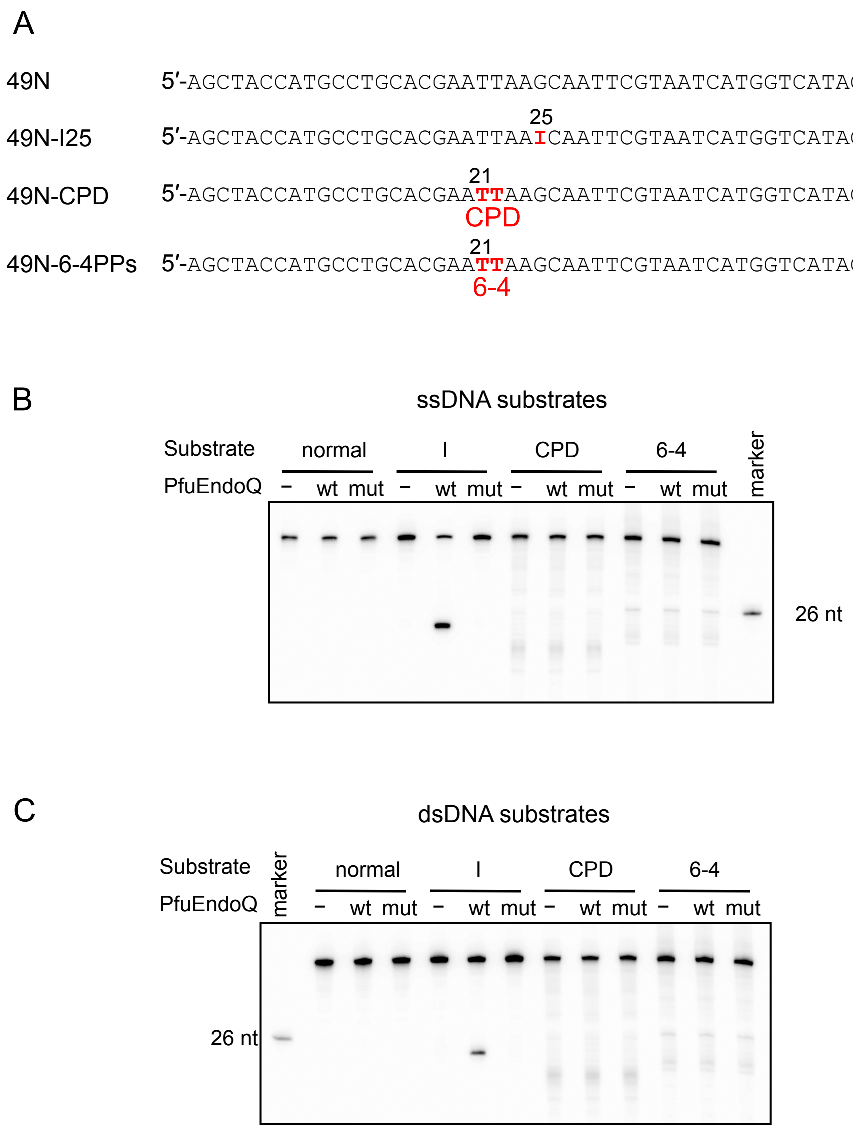

***Figure S5.* Nuclease activity of PfuEndoQ against UV-damaged DNA.** (A) The nucleotide sequences of the substrates containing cyclobutane pyrimidine dimer (CPD), pyrimidine (6-4) pyrimidone photoproducts (6-4 PPs), and dI. (B, C) Nuclease activity of PfuEndoQ (wt) or its inactivated mutant, PfuEndoQ D193A (mut), on ssDNA substrate (B) and dsDNA substrates (C). The reaction mixtures containing 10 nM PfuEndoQ and 10 nM ^{32}P-labeled substrate were incubated in the solution with 50 mM TrisHCl, pH 8.0, 50 mM NaCl, 1 mM $MgCl_2$, and 0.1% Tween 20 at 75˚C for 10 min (ssDNA) and at 65˚C for 60 min (dsDNA). The reaction mixtures were analyzed by 8M Urea-15% PAGE followed by autoradiography using Typhoon Trio+ Imager (GE Healthcare).

A

ssDNA	5′Cy5-GGAATCCTGACGACITGTAGCGAACGATCA
dsDNA	5′Cy5-GGAATCCTGACGACITGTAGCGAACGATCA<br>3′-CCTTAGGACTGCTGTACATCGCTTGCTAGT
DNA/RNA	5′Cy5-GGAATCCTGACGACITGTAGCGAACGATCA<br>3′-CCUUAGGACUGCUGUACAUCGCUUGCUAGU
ssRNA	5′Cy5-GGAAUCCUGACGACIUGUAGCGAACGAUCA
dsRNA	5′Cy5-GGAAUCCUGACGACIUGUAGCGAACGAUCA<br>3′-CCUUAGGACUGCUGUACAUCGCUUGCUAGU
RNA/DNA	5′Cy5-GGAAUCCUGACGACIUGUAGCGAACGAUCA<br>3′-CCTTAGGACTGCTGTACATCGCTTGCTAGT
marker	5′Cy5-GGAAUCCUGACGACIU

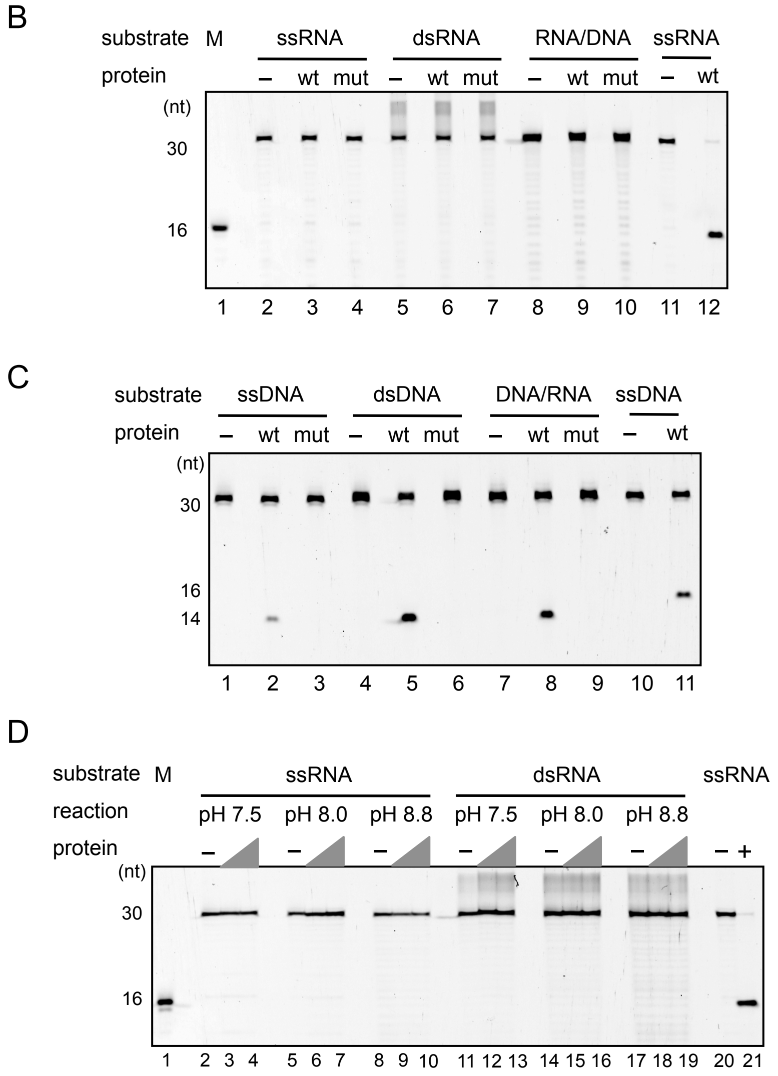

Figure S6. **Nuclease activity of PfuEndoQ against RNA.** (A) The nucleotide sequences of the substrates. (B, C, and D). Various substrates containing inosine were incubated with PfuEndoQ (wt) or its mutant PfuEndoQ D193A (mut). The reaction mixtures containing 10 nM PfuEndoQ and 10 nM Cy5-labeled substrate were incubated in the solution with 50 mM TrisHCl, pH 8.0, 50 mM NaCl, 1 mM $MgCl_2$, and 0.1% Tween 20 at 75°C for 10 min. The reaction mixtures were analyzed by 8 M urea, 15% PAGE, and the Cy5-labeled oligonucleotide was visualized by Typhoon Trio+ Imager (GE Healthcare). (B) Cy5-labeled RNA substrates were used. Lane 1, product marker (16 nt); lanes 2-10 PfuEndoQ; lane 12, PfuEndoV (positive control). (C) Cy5-labeled DNA substrates were used. Lanes 1-9, PfuEndoQ; lane 11, PfuEndoV (positive control). (D) Cy5-labeled RNA substrates were incubated with PfuEndoQ (10 nM or 50 nM) in the solutions of various pH. Lane 1, marker (16 nt); lanes 2-10, ssRNA incubated with PfuEndoQ; lanes 11-19, dsRNA incubated with PfuEndoQ; lane 21 Pfu EndoV was used as a positive control.
